# Prevalence of exclusive breastfeeding practice and associated factors among mothers having infants less than 6 months old, in Bahir Dar, Northwest, Ethiopia: a community based cross sectional study, 2017

**DOI:** 10.1186/s13104-018-3877-5

**Published:** 2018-10-29

**Authors:** Amare Belachew, Tilahun Tewabe, Adino Asmare, Desta Hirpo, Banchialem Zeleke, Desalegn Muche

**Affiliations:** Department of Nursing, College of Medicine and Health Science, Bahir Dar University, Bahir Dar, Ethiopia

**Keywords:** Exclusive breastfeeding practice, Prevalence, Bahir Dar, Northwest Ethiopia

## Abstract

**Background:**

Breast milk provides all the energy and nutrients that the infant needs for the first 6 months of life. Suboptimal breastfeeding especially lacks exclusive breastfeeding increase risk of severe acute malnutrition by 3.2-fold and major contributory factor for infant child mortality. Therefore, the objective of this study was to assess the prevalence of exclusive breastfeeding practice and associated factors among mothers having infants less than 6 months old in Bahir Dar city, Northwest, Ethiopia, 2017.

**Result:**

The prevalence of exclusive breastfeeding practice 1 day before the survey was 86.4%. Mothers who; have young infant aged 0–1 month old [AOR = *5.702 (1.747, 18.613*)], house wife [AOR = *2.995 (1.557, 5.690)*] and are not influenced by culture [AOR = *11 (3.449, 35.165)*] were more likely to practice exclusive breastfeeding than their counterparts.

## Background

Exclusive breastfeeding means giving infants only breast milk and no other liquids or foods with the exception of vitamins, minerals, and medicines for the first 6 months of infant’s life [1]. Breast milk is a natural food that serves as a complete source of infant nutrition for the first 6 months of life. It has all components of nutrients and minerals which protects both the mothers and the child against illnesses and diseases with its immunological property [2].

Exclusive breastfeeding for the first 6 months of life can reduce around 13% of infant mortality [2]. Breastfeeding and exclusive breastfeeding in a particular, is one of the measurement strategies to improve infant’s nutritional status and survival. Every year, around 10 million deaths of children younger than 5 years old are caused by due to the direct or indirect consequence of the malnutrition [3]. Moreover, a 3.2-fold increased risk of severe acute malnutrition is observed in non exclusively breastfed children compared to those who are exclusive breastfeeding. This is the reason why WHO and UNICEF have formulated the global recommendation of optimal breastfeeding for infants less than 6 months old and continued up to 2 years of age [2, 4, 5]. In the developing world, more than 40% of the infants under 6 months old are benefited from the practice of exclusive breastfeeding and these is particularly low in Africa, where less than one third of the infants under 6 months of age are exclusively breastfeeding [6].

In Ethiopia, nearly 321,000 under five children died in each year from which malnutrition is the cause for 57% deaths primarily through the exacerbation of other major causes, such as diarrhea and pneumonia, which can be easily prevented through exclusive breastfeeding [7, 8]. Currently, the practice of exclusive breastfeeding in Ethiopia is low, and according to EDHS, 2016 report, only 58% of mothers with infants less than 6 months old breastfeed them exclusively [9]. Unfortunately, for all the recognized advantages and efforts deployed to promote exclusive breastfeeding; the practice of exclusive of breastfeeding among 2–3 months old infants were 64% and it decreased to 36% when infant’s age increased to 4–5 months [9].

Even though the government of Ethiopia developed the infant and young child feeding guideline, there is the varying level of intervention were being given both at the community level and health institutions. The efforts were not organized at the level of practice and this is due low number of studies to explore exclusive breastfeeding in Ethiopia. Therefore, the aim of this study was to assess the prevalence of exclusive breastfeeding practice and associated factors among mother having infants less than 6 months old in Bahir Dar city, Ethiopia.

## Methods

### Study setting and period

A community based cross sectional study was conducted in Bahir Dar city from April 15 to May 3, 2017. Bahir Dar is the capital city of Amhara regional state. It is 565 km away from Addis Ababa which is the capital city of Ethiopia. The estimated population of the city is 221,991 among them 49% were males and 51% were females. The city has nine sub cities, one public specialized referral hospital, one district hospitals, two private hospitals, ten health centers, nine health posts, private clinics, and private pharmacy which gives serves to the town and the surrounding people.

### Sample size and sampling techniques

The sample size was determined by using single population proportion formula with considering the following assumptions: prevalence (P) = 82% proportion of exclusive breastfeeding practice in Ambo [10], confidence level (Cl) = 95%, margin of error (W) = 5%, and by using design effect of two (Bahir Dar city—sub cities—kebeles-households) and after adding 10% non response rate the final sample size was 499. Bahir Dar city has nine sub cities and out of them three sub cities were selected by using a lottery method. In the selected sub cities, nine kebeles with a total of 1497 households having less than 6 months old infants were taken from local health extension workers. Finally, 499 households were selected by simple random sampling technique i.e. lottery method. Reviewing the birth date certificate and asking the mothers were used to determine the actual age of infants.

### Measurement

Data were collected using an interviewer administered questionnaire. The questionnaire was adopted from previous researches done on similar topics [9, 11, 12] and modifying accordingly. A 1 day (24 h) infant diet recall method was used to assess exclusive breastfeeding. Training was given for data collectors and supervisors for 2 days on methods of extracting the information through interviewing, how to fill the information on a structured questionnaire and the ways of approaching to the respondents.

### Statistical analysis

The data was cleaned, coded, entered into Epi Data software version 3.5.4 and transferred into SPSS version 20 for analysis. Descriptive statistics were used to describe the distributions of variables. Logistic regression was used to assess the relation between dependent and predictor variables. First bivariate analysis was done to examine the associations of single independent variable with exclusive breastfeeding practice. Independent variables with a P-value ≤ 0.05 in bivariate analysis were entered into the multivariate analysis. Association between dependent and predictor variables was assessed using AOR and 95% CI. Statistical significance was declared when the P-value was less than 0.05.

### Operational definition

Exclusive breastfeeding practice: If a mother feed only breast milk to her infant 1 day (24 h) before the survey conducted [9, 11].

## Result

### Socio-demographic characteristics

From all 499 eligible mothers, 472 were participated in this study which made a response rate of 94.6%. One hundred sixty eight (34.5%) mothers were between the age of 25–29 years, 96.4% were Amhara by ethnicity, 75.2% were orthodox Christian, 22.9% attend primary education, 53.45% were house wives by occupation and 40.5% mothers earn greater than 300 ETB per month. More than a half (63.1%) of mothers lived in a nuclear family. More than half (59.3%) of children were between 91–180 days of age. Regards infant sex, 54.7% were females (Table 1).

**Table 1:** Socio-demographic characteristics of mothers (respondents) who have infants less than 6 months old, in Bahir Dar town, North West, Ethiopia, 2017

Variable	Category (n = 472)	Frequency	Percent (%)
Infant sex	Male	214	45.3
	Female	258	54.7
Infant age	0–30 days	71	15
	31–90 days	140	29.7
	91–180 days	261	55.3
Age of mothers	15–19	30	6.4
	20–24	119	25.2
	25–29	163	34.5
	30–34	98	20.8
	35 and above	62	13.1
Religion	Orthodox	355	75.2
	Muslim	94	20
	Protestant	12	2.5
	Catholic	11	2.3
Ethnicity	Amhara	455	96.4
	Tigre	6	1.3
	Oromo	6	1.3
	Others	5	1
Level of education of mothers	No education	86	18.2
	Able to read and write	55	11.7
	Primary school (1–8)	108	22.9
	Secondary	86	18.2
	Higher	137	29
Occupational status of mother’s	Housewife	252	53.4
	Governmental employee	108	22.9
	Private employee	46	9.7
	Merchant	38	8.1
	Daily labor	28	5.9
Marital status of mother	Single	36	7.6
	Married	409	86.7
	Widowed	6	1.3
	Divorced	21	4.4
Husband educational level (409)	No education	30	7.3
	Able to read and write	51	12.5
	Primary school (1–8)	103	25.1
	Secondary	85	20.80
	Higher	140	34.3
Husband occupation (409)	Govt employee	118	28.9
	Private employee	77	18.8
	Merchant	116	28.4
	Daily labor	64	15.6
	Farmer and driver	34	8.3
Type of family	Nuclear family	298	63.1
	Extended family	174	36.9
Household income	≤ 1000	59	12.5
	1001–2000	160	33.9
	2001–3000	62	13.1
	> 3000	191	40.5
ANC follow up	Yes	370	78.4
	No	102	21.6
Ways of delivery	Normal	287	60.8
	C/s	185	39.2
Place of delivery	Home	119	25.2
	Health institution	353	74.8

#### Exclusive breast feeding practice

About 86.4% of mothers exclusively breastfeed their infant 1 day before the survey. Majority of mothers (75.4%) initiated breastfeeding within 1 h. Three hundred ninety six (83.9%) infants fed colostrum and 74.6% infants were not fed prelacteal feeding. The main reasons of mothers for not practicing exclusive breastfeeding were; shortage of time to practice EBF (15.6%) and perceived as human milk is not sufficient for their infant (27%) (Table 2).

**Table 2:** Breastfeed and related practice among mothers who have infant less than 6 months, in Bahir Dar city, North West, Ethiopia, 2017 (n = 472)

Variable	Frequency	Percent (%)
Breastfeeding experience of current infant		
Yes	461	97.7
No	11	2.3
Timely initiation of breastfeed within 1 h		
yes	356	75.4
No	116	24.6
Colostrums feeding		
Yes	396	83.9
No	76	16.1
Prelacteal feeding		
Yes	120	25.4
No	352	74.6
Infant feeding 1 day before survey		
Exclusively BF	408	86.4
In addition to breast milk water, sugar solution, tea, soft foods	64	13.6
Reasons for not exclusively BF (64)		
Culture/tradition	17	26.7
Not sufficient for infant	19	29.7
Infant becomes thirsty	5	7.8
Lack of time/work over load	12	18.7
Maternal illness	9	14.1
Lack of information’s	2	3
Who influence you to give other feeding (64)		
Husband	29	45.3
My mother	18	28
Mother in law	10	15.6
Health worker	4	6.3
My own decision	2	3.2
Knowledge about breastfeeding practice		
BF is important for infant health		
Yes	472	100
No	0	
BF is important for maternal health		
Yes	324	68.6
No	148	31.4
Put immediately after birth		
Yes	422	89.4
No	50	10.6
Colostrums should be given to an infant		
Yes	423	89.6
No	49	10.4
Prelacteal feeding is needed before start BF		
Yes	62	13.1
No	410	86.9
EBF for 6 months		
Yes	423	89.6
No	49	10.4
Infants start complementary feeding at 6 month and continue BF up to 2 years and beyond		
Yes	436	92.4
No	36	7.6

Majority of mothers (89.4%) know as the infant should be put on breastfeeding immediately within 1 h, 89.6% know as breast milk is enough without water and other liquids for the first 6 months (Table 2).

### Factors associated with exclusive breastfeeding practice

In bivariate analysis, the factors found to be significantly associated with exclusive breastfeeding practice were; Infant age, occupational status of mother, colostrums feeding, maternal age and cultural influences.

Finally, mother of infant age 0–1 month old, unemployed mother and mothers who are not influenced by culture was the predictor of exclusive breastfeeding.

Age of infant was significantly associated with exclusive breastfeeding practice. Mothers with young infant (aged 0–1 month old) were 5.7 times more likely to practice exclusive breastfeeding than mothers with infant aged 4–6 months old [AOR = *5.702 (1.747, 18.613)*].

Mother’s occupational status was a determinant factor for exclusive breastfeeding practice. House wife mothers were around three times more likely to practice exclusive breastfeeding than mothers having additional duties or jobs [AOR = *2.995 (1.557, 5.690)*].

Mother’s traditional or cultural belief towards initiation of first breast milk was a significant factor for exclusive breastfeeding practice. Mothers who were not influenced by culture/belief towards breast milk were 11 times more likely to practice exclusive breastfeeding than mother influenced by culture/belief [*AOR = 11 (3.449, 35.165)*] (Table 3).

**Table 3:** Factors that affect EBF practice among mothers of infants age less than 6 months using bivariate and multivariate logistic regression analysis model, Bahir dar, Ethiopia, 2017

Variables	EBF practice				
		Yes (n & %)	No (N & %)	COR (95% CL)	AOR (95% CL)
Age of child in months	0–1	67 (94.4)	4 (5.6)	3.679 (1.278,10.58)	5.702 (1.747, 18.613)*
	2–3	127 (90.7)	13 (9.3)	2.146 (1.118, 4.119)	2.778 (1.279, 6.032)*
	4–6	214 (82)	47 (18)	1	1
Occupational status	Unemployed	229 (90.9)	23 (9.1)	2.281 (1.321, 3.940)	2.995 (1.557, 5.690)*
	Employed	179 (81.4)	41 (18.6)	1	1
Colostrums feeding	Yes	345 (88.5)	45 (11.5)	2.706 (1.455, 5.033)	.899 (0.379, 2.133)
	No	51 (73.9)	18 (26.1)	1	1
Maternal age	15–29 years	262 (84)	50 (16)	1	
	≥ 30 years	146 (91.2)	14 (8.8)	1.99 (1.064, 3.723)	1.664 (0.833,3.323)
Cultural influence	Yes	10 (58.8)	7 (41.2)	1	1
	No	398 (87.5)	57 (12.5)	4.88 (1.789, 13.352)	11.01 (3.449, 35.16)*

1 = reference, * = *p* value less than 0.05, N = number, % = percent

## Discussion

EBF is recommended in the first 6 months of infant’s life. The prevalence of exclusive breastfeeding practice 1 day before the survey conducted in the study area was 86.4%. This result is comparable with studies done in Ambo 82.2% [10], Sri Lanka 85% [13], Dubti town, afar regional state 81.1% [14], and near to WHO recommendations 90% by 2020. This finding is greater than studies done in; Motta 50.1% [11], 2011 Ethiopian EDHS report 52% [9], East Gojjam, Gozamin district 74.1% [15], Debremarkos 60.8% [16], SNNPR 64.8% [17] and Goba district 71.3% [12]. This may be due to differences in maternal socio demographic characteristics like educational status of mothers and house hold income, culture/belief, awareness of mother and commitments of community health extension workers in the study area and other studies.

Mother with young infant aged 0–1 month old was 5.7 times more likely to practice exclusive breastfeeding than mother with infant aged 4–6 months old. This result is consistent with finding in; Motta [11], Debre Markos [16], Gozamin District [15], Debre Tabor [18], Bale Goba [12] and Djibouti [14]. This may be due to mother perception that; breast milk is not sufficient as age of the infant increases makes the infant thirsty of water and they perceived that giving water or other liquids in addition to breast milk alleviates abdominal cramps of the infant.

Occupational status of mother was associated with exclusive breastfeeding practice. House wife mothers were around three times more likely to practice exclusive breastfeeding than mothers having additional jobs outside the home. This finding is similar with studies done in Ethiopia; Ambo [10], Motta [11], Gozamen district [15], Gobba district [12] and Awi Zone [19]; similarly this study is also consistent with studies done in Ghana [20], Netherlands [21] and Cameroon [22]. This may be due to employed mothers; passed more than 8 h on work, lack private room for breastfeeding, remoteness of the work area from the home, lack of organizational support, short period of maternity leave, inflexible work schedule and low availability of day care centers.

Mother’s traditional or cultural belief was the determinant factor for exclusive breastfeeding practice. Mothers who were not influenced by culture/tradition were 11 times more likely to practice exclusive breastfeeding than those mother who were influenced by culture/tradition. This finding is consistent with studies done in Kenya [23] and Hindus [24]. This is may be due to the false perception of mother’s i.e. first milk as dirty milk and transmit abdominal cramps to their infants. Additionally, mother of the infant may believe that they inherently unable to produce enough milk when their ancestors were not produce enough milk.

## Conclusion

Majority of mothers practiced exclusive breastfeeding. Infant age, mother occupation, and traditional or cultural influences were the determinant factors for exclusive breastfeeding practice. Recommendations to increase EBF were: health extension workers who are working, participating and educating the community should change the false perception of mothers, family, and the community as a whole on breastfeeding and related traditional practices like milking and throwing colostrums, giving water to relieve thirsty and abdominal cramp and early introduction of complementary feeding.

## Limitations

The study did not assessed qualitative aspects of exclusive breastfeeding Using a 24 h diet recall method may overestimated the magnitude of exclusive breastfeeding.

## Abbreviations

BFHI: Babies friendly Hospital Initiation
DHS: Demographic Health Survey
EBF: exclusive breast feeding
ICMBMS: International Code of Marketing Brest Milk Substitute
IYCF: infant and young child feeding
UNICEF: United Nation International
WHO: World Health Organization
